# Supplementary material for: Isolation and Characterization of Extracellular Vesicles from Gastric Juice
Source: Cancers (Basel). 2022 Jul 7;14(14):3314. doi: 10.3390/cancers14143314 (PMC9318556; doi:10.3390/cancers14143314)
Supplement: Supplementary file 1 [file cancers-14-03314-s001.zip › cancers-1768153-supplementary.pdf]

cancers

MDPI

Supplementary Materials: Isolation and Characterization of Extracellular Vesicles from Gastric Juice

Gleb O. Skryabin, Svetlana V. Vinokurova, Sergey A. Galetsky, Danila S. Elkin, Alexey M. Senkovenko, Darya A. Denisova, Andrey V. Komelkov, Ivan S. Stilidi, Ivan N. Peregorodiev, Olga A. Malikhova, Oiatiddin T. Imaraliev, Adel D. Enikeev and Elena M. Tchevkina

Upper part

Alix

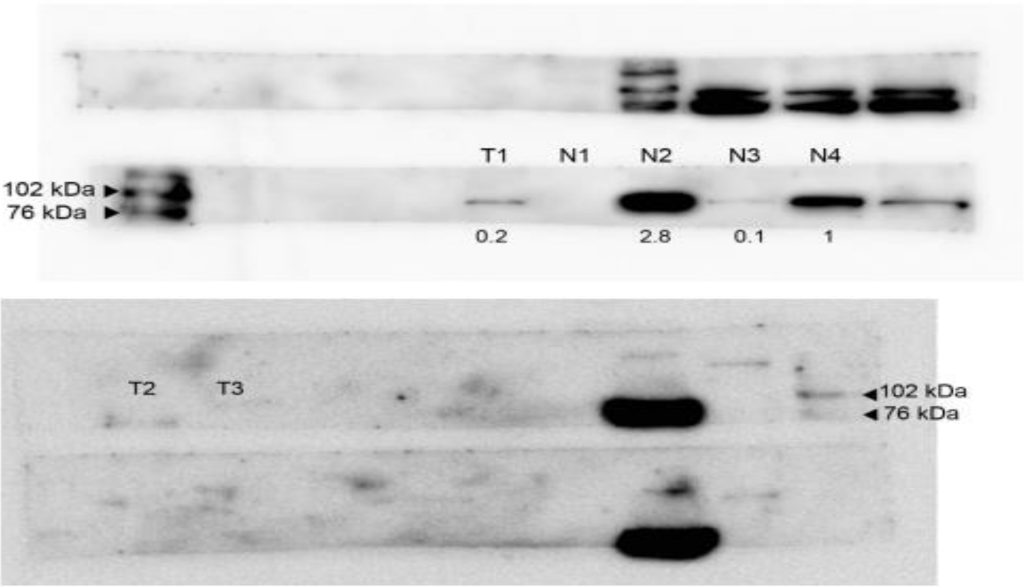

Flot-2

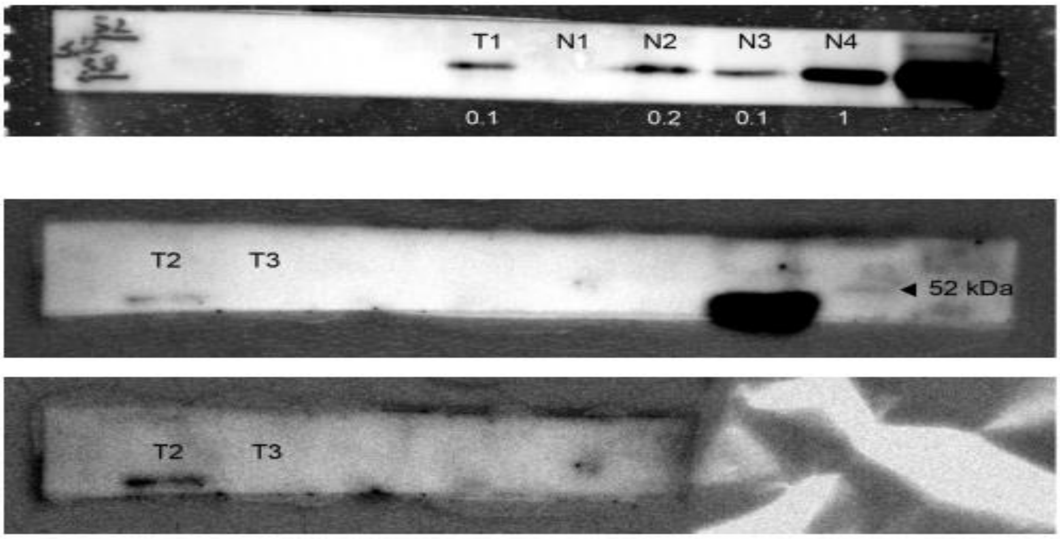

TSG-101

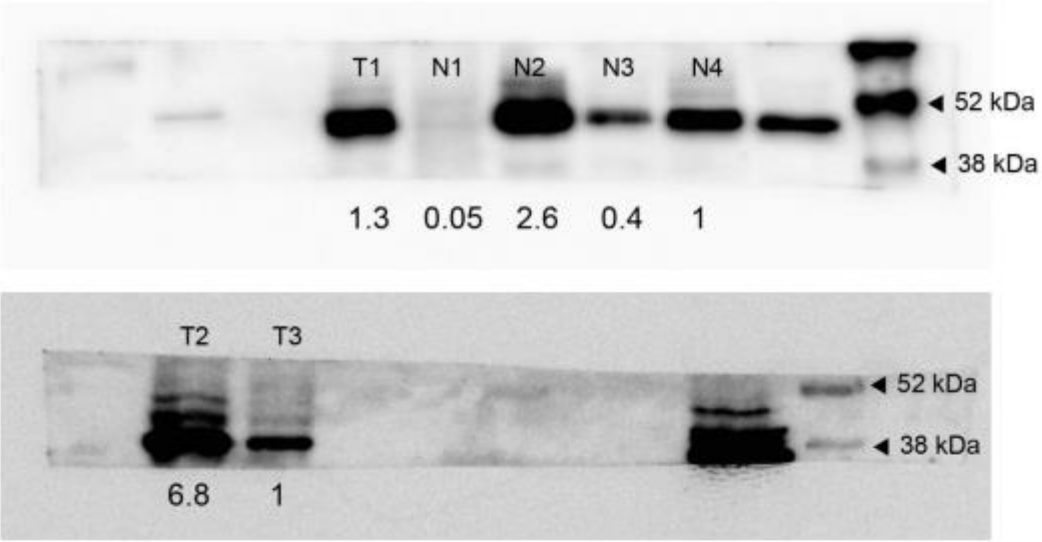
T1 N1 N2 N3 N4
52 kDa
38 kDa
1.3 0.05 2.6 0.4 1
T2 T3
52 kDa
38 kDa
6.8 1

Stom

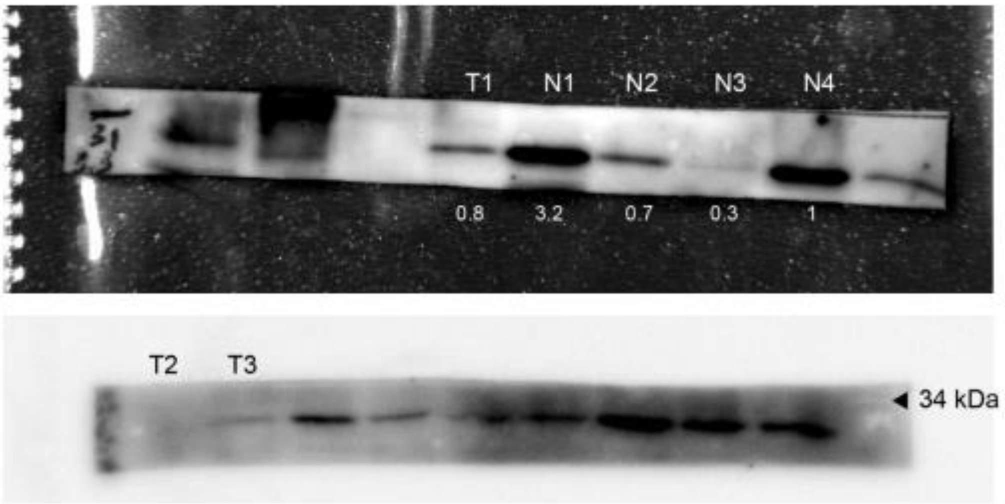
T1 N1 N2 N3 N4
0.8 3.2 0.7 0.3 1
T2 T3
34 kDa

CD9

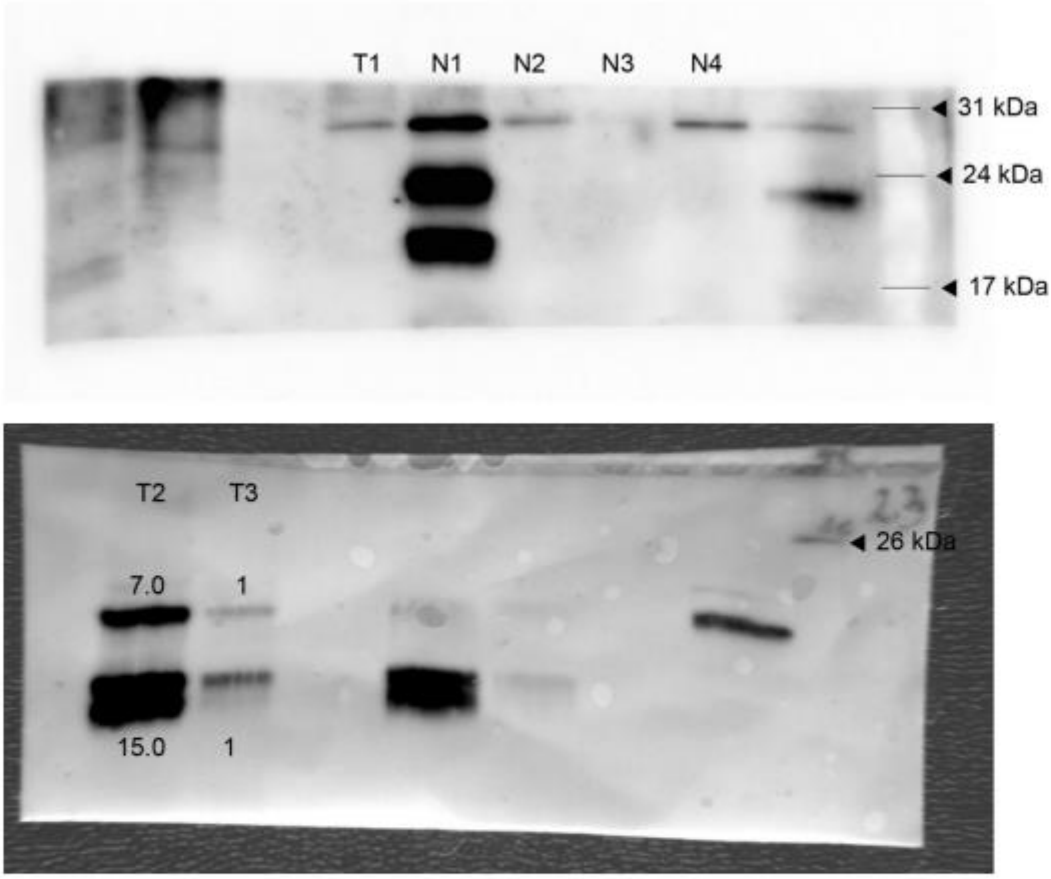

Lower part

Alix

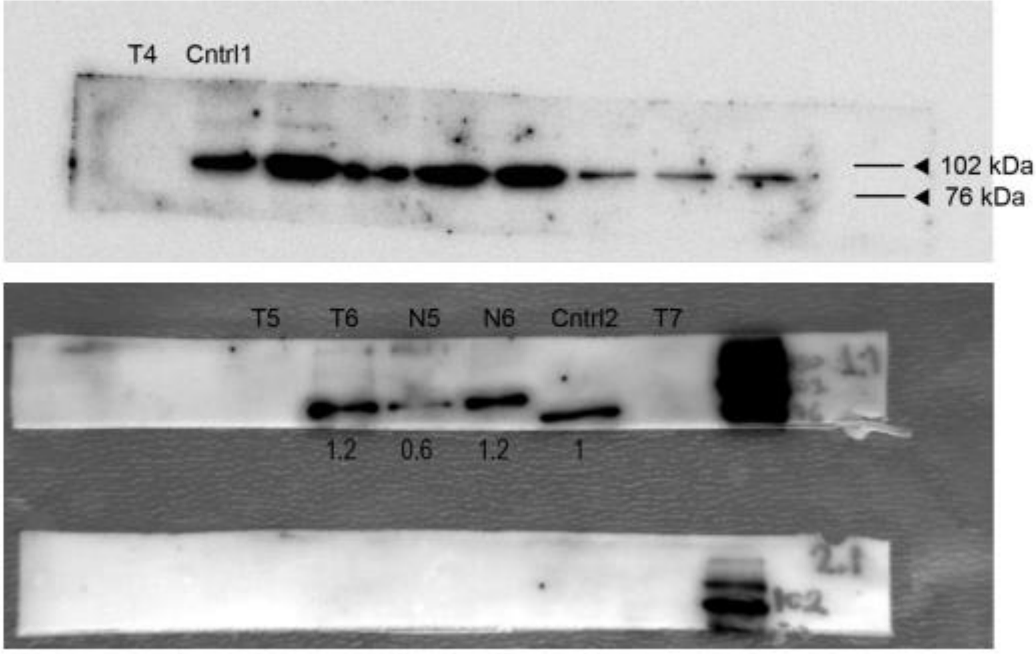

Flot-2

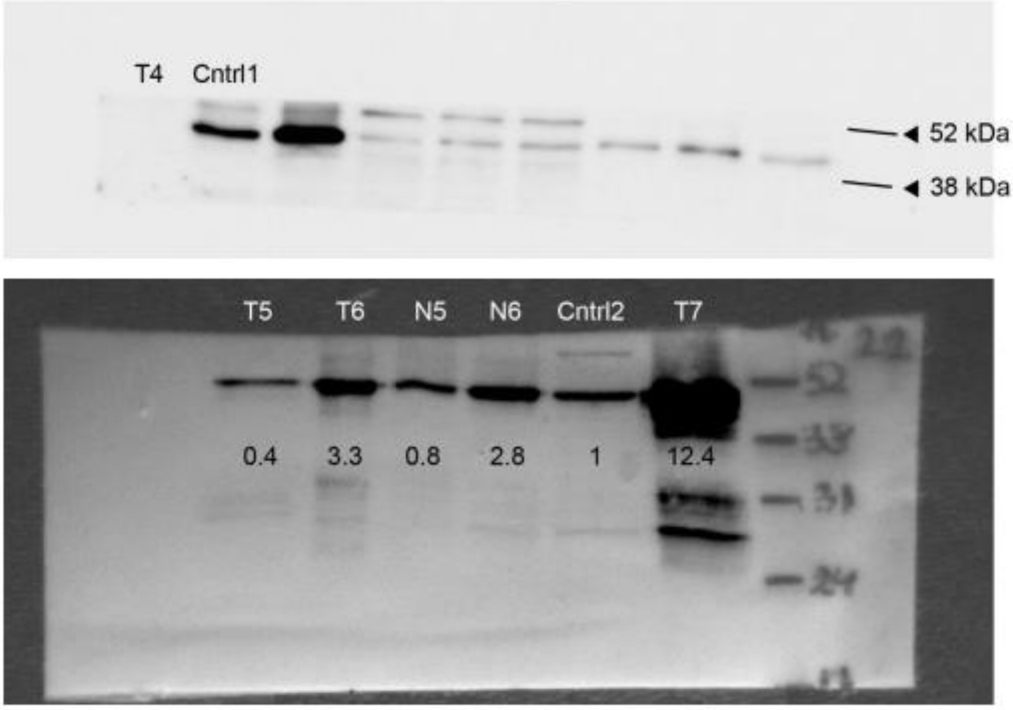

TSG-101

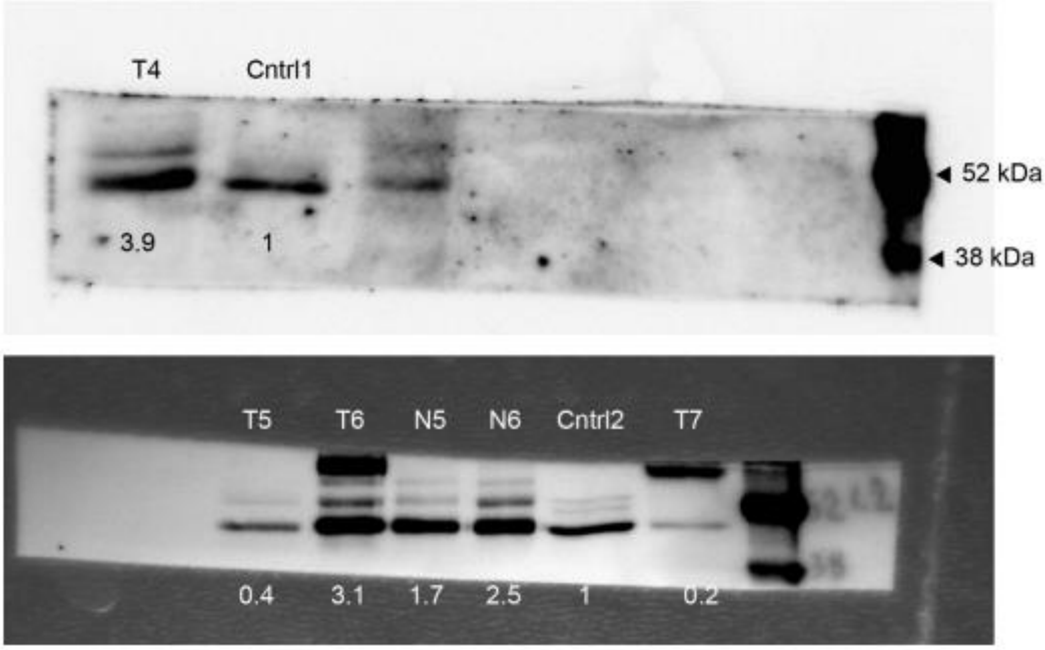

Stom

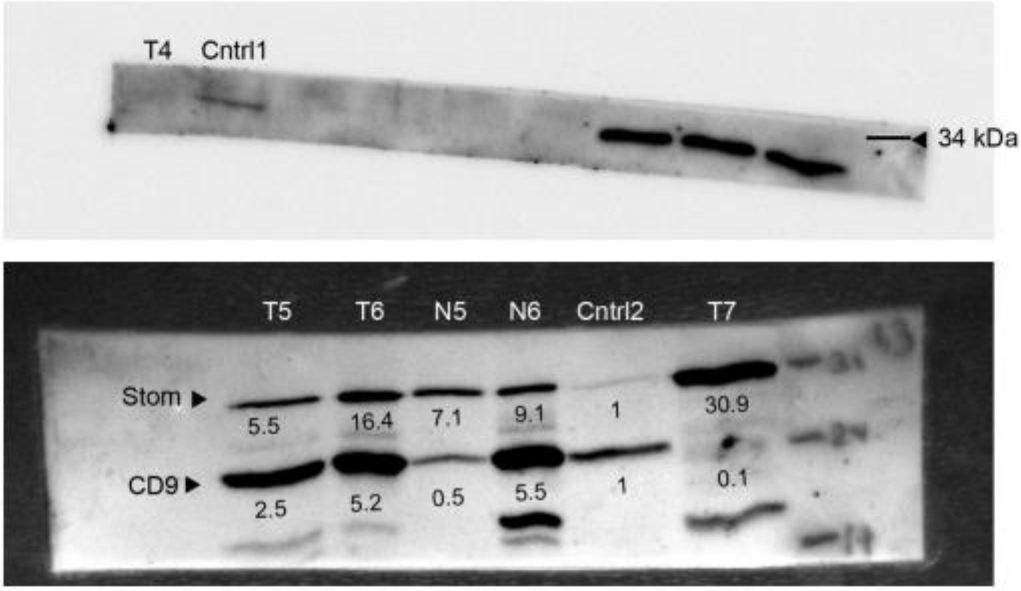

CD9

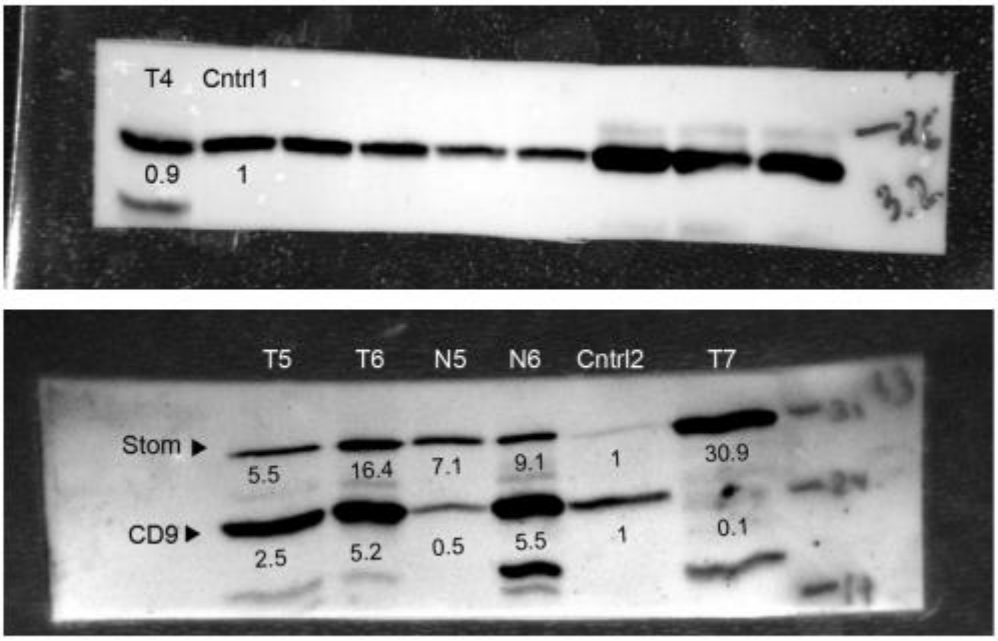

PCNA

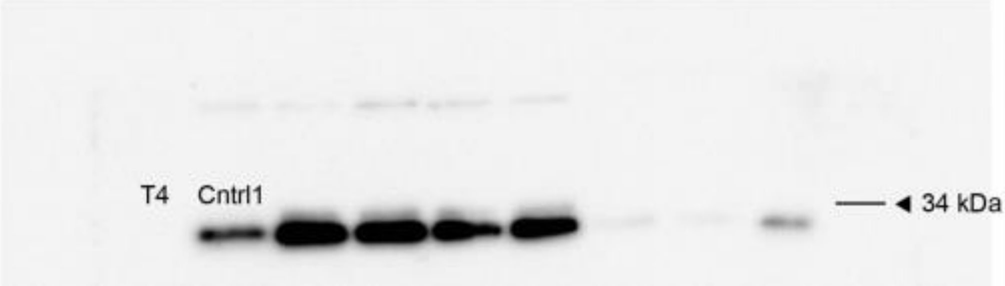

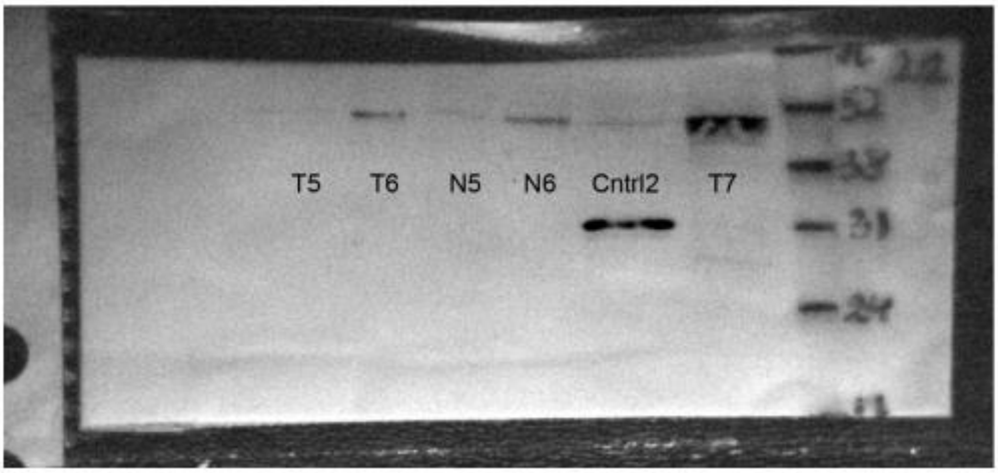

Figure S1. Full Western Blot images from Figure 2A.

Table S1. Clinical and morphological characteristics of GC patients and NTA data on EV size distribution and concentration.

Clinical and MorphologicalCharacteristics			NTA Data											
Sample	Histotype	Grade	Mean, nm		Mode, nm		Median, nm		10th percentile, nm		90th percentile, nm		Conc. × 10^{12} particles per mL	
			Value	SD	Value	SD	Value	SD	Value	SD	Value	SD	Value	SD
T1	adenocarcinoma	high	99	9	58	3	73	4	38	2	197	27	3.30	0.34
T2	adenocarcinoma	high	172	8	141	13	163	13	65	4	309	23	6.61	1.24
T3	adenocarcinoma	high	170	9	128	25	164	13	65	5	299	30	15.75	3.70
T4	adenocarcinoma	low	148	8	123	21	140	16	60	6	263	23	4.38	0.57
T5	adenocarcinoma	low	154	6	93	11	143	15	57	3	275	16	8.54	1.22
T6	adenocarcinoma	high	153	14	83	34	140	23	58	7	275	38	0.48	0.12
T7	adenocarcinoma	low	159	16	103	46	140	20	57	5	300	45	3.06	0.23
N1	-	-	178	7	116	39	171	17	68	3	318	14	7.63	0.57
N2	-	-	151	4	63	11	128	8	47	4	290	6	5.19	0.74
N3	-	-	92	8	57	8	62	2	36	1	213	22	1.85	0.21
N4	-	-	122	6	52	5	94	11	39	1	245	21	2.77	0.12
N5	-	-	176	15	91	23	166	24	62	4	322	34	4.92	0.70
N6	-	-	169	10	66	26	146	17	56	2	320	35	3.18	0.53

Table S2. Sequences of reverse transcription primers used; sequences of RT-qPCR primers andTaqMan™ probes used.

	Target	Sequence 5′–3′
Reverse Transcription primers	miR-199a-3p RT	GTCGTATCCAGTGCAGGGTCCGAGGTATTCGCACTGGATACGACtaacca
	miR-204-3p RT	GTCGTATCCAGTGCAGGGTCCGAGGTATTCGCACTGGATACGACaggatc
	miR-451a RT	GTCGTATCCAGTGCAGGGTCCGAGGTATTCGCACTGGATACGACaactcag
	miR-23a-3p RT	GTCGTATCCAGTGCAGGGTCCGAGGTATTCGCACTGGATACGACggaaatc
	let-7b-5p RT	CTCAACTGGAGCTAGTTTCGTCGTAGGGCAGTTGAGAACCACAC
	miR-16-5p	GTTGGCTCTGGTGCAGGGTCCGAGGTATTCGCACCAGAGCCAACcgccaa
PCR Reverseprimer	Uni-Ch-R	GTGCAGGGTCCGAGGT
	R1 (only for let-7b)	CTGGAGCTAGTTTCGTCGTAG
PCR Forwardprimers	miR-199a-3p F	CAGCTGGGACAGTAGTCTGC
	miR-204-3p F	CACGCAGCTGGGAAGGCAA
	miR-451a F	CACGCATAAACCGTTACCA
	miR-23a-3p F	ATCACATTGCCAGGGATT
	let-7b-5p F	CAGCTGGGTGAGGTAGTAG
	miR-16-5p F	GTTTGGTAGCAGCACGTAAATA
PCR TaqManprobes with LNA modifications	miR-199a-3p P	**FAM**-CATTGGTTAGTCGTATC-**BHQ1**
	miR-204-3p P	**FAM**-GGGACGTGTCGTATCCAG-**BHQ1**
	miR-451a P	**FAM**-TTACTGAGTTGTCGTATCC-**BHQ1**

miR-23a-3p P	**FAM**-CGCACTGGATACGACGGAAATCC-**BHQ1**
let-7b-5p P	**HEX-**GTTGTGTGGTTCTCAACTG-**BHQ1**
miR-16-5p P	**FAM**-TTGGCGGTTGGCTCTG-**BHQ1**

Table S3. Average Ct values of hsa-miR-199a-3p, hsa-miR-204-3p, hsa-miR-451a, hsa-miR-23a-3p, hsa-miR-16-5p, has-let-7b-5p obtained by RT-qPCR in EVs isolated from gastric juice of patients suffering from gastric cancer and non-cancer individuals.

	**miR-199a-3p**	**miR-204-3p**	**miR-451a**	**let7b-5p**	**miR-16-5p**	**miR-23a-3p**	**miR- 135b-5p**	**miR- 135b-3p**
N1	35.03	33.60	31.94	31.13	28.54	26.15	31.06	39.04
N3	34.78	31.56	32.73	31.35	31.20	25.47	34.00	37.96
N4	37.79	34.66	35.29	34.42	32.58	26.41	34.41	40.20
N2	34.63	30.53	32.33	32.89	33.11	26.55	32.97	38.54
N5	36.09	35.10	33.85	32.33	32.47	26.23	32.01	40.19
N6	36.38	36.03	35.14	34.27	34.04	26.71	34.78	42.74
T7	33.54	34.46	35.59	30.19	27.65	27.27	30.98	37.17
T5	36.29	34.18	36.61	33.86	35.04	26.79	35.24	41.75
T6	36.35	33.78	33.87	32.12	31.85	26.38	31.40	36.69
T1	35.93	34.93	38.01	36.19	36.28	26.56	38.15	39.05
T2	32.71	31.54	33.49	33.00	31.57	25.53	37.77	36.14
T3	31.91	32.79	34.98	31.13	28.56	25.58	31.12	36.86
T4	32.89	32.48	36.89	33.75	30.25	26.31	34.84	37.53
